# Ambient ionisation mass spectrometry for in situ analysis of intact proteins

**DOI:** 10.1002/jms.4087

**Published:** 2018-05-24

**Authors:** Klaudia I. Kocurek, Rian L. Griffiths, Helen J. Cooper

**Affiliations:** ^1^ School of Biosciences University of Birmingham Edgbaston Birmingham B15 2TT UK

**Keywords:** ambient mass spectrometry, surface sampling, intact proteins, ion mobility spectrometry, LESA, nanoDESI, DESI, Flowprobe

## Abstract

Ambient surface mass spectrometry is an emerging field which shows great promise for the analysis of biomolecules directly from their biological substrate. In this article, we describe ambient ionisation mass spectrometry techniques for the in situ analysis of intact proteins. As a broad approach, the analysis of intact proteins offers unique advantages for the determination of primary sequence variations and posttranslational modifications, as well as interrogation of tertiary and quaternary structure and protein‐protein/ligand interactions. In situ analysis of intact proteins offers the potential to couple these advantages with information relating to their biological environment, for example, their spatial distributions within healthy and diseased tissues. Here, we describe the techniques most commonly applied to in situ protein analysis (liquid extraction surface analysis, continuous flow liquid microjunction surface sampling, nano desorption electrospray ionisation, and desorption electrospray ionisation), their advantages, and limitations and describe their applications to date. We also discuss the incorporation of ion mobility spectrometry techniques (high field asymmetric waveform ion mobility spectrometry and travelling wave ion mobility spectrometry) into ambient workflows. Finally, future directions for the field are discussed.

## 1 INTRODUCTION

The interest in proteins is fuelled by their fundamental role in the biological processes occurring in living cells, both under normal conditions and in disease states. A key advantage of the analysis of proteins in their intact form is that all information relating to primary structure and posttranslational modifications is retained. Whereas the presence of single amino acid substitutions, or connectivity between posttranslational modifications, may be lost in the analysis of enzymatic digests of proteins, that is not possible when interrogating the intact protein. Moreover, by considering intact proteins, it is possible to probe their tertiary (and quaternary) structures and interactions. The desire to characterise the structures of proteins is coupled with a growing demand for information on their spatial distribution within tissues. Over the last 40 years, a suite of mass spectrometric techniques have been developed which are capable of achieving all of these objectives. Ambient methods are especially useful in that they do not generally require any prior sample preparation or disruption, preserving much more biologically relevant information than vacuum techniques. Particularly noteworthy is the emerging field of native ambient mass spectrometry, ie, the development of approaches which preserve the tertiary and quaternary structure of proteins.

In this feature, we describe surface analysis techniques most commonly used for the mass spectrometric analysis of intact proteins, with emphasis on liquid extraction‐based techniques which, thus far, have proven most effective for intact protein analysis directly from biological substrates. We present their advantages and current limitations, as well as their applications to date, focusing on in situ intact (top‐down) protein analysis from biological surfaces such as tissue sections, dried blood spots, and bacterial communities. We also provide a brief overview of the potential future developments.

## 2 AMBIENT SURFACE SAMPLING MASS SPECTROMETRY TECHNIQUES

**Figure 1:**
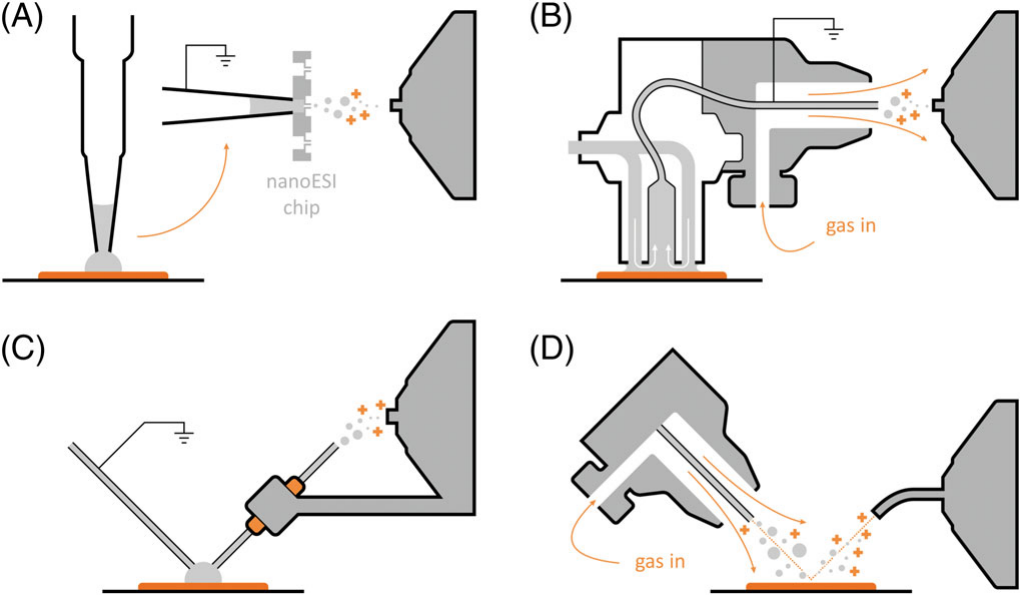
Ambient ionisation techniques for protein analysis. A, Liquid extraction surface analysis. B, Flowprobe. C, Nano desorption electrospray ionisation. D, Desorption electrospray ionisation

Arguably, the most important development enabling the analysis of macromolecules was the invention of electrospray (ESI).^1^ All ambient surface sampling techniques make use of electrospray ionisation in some form.^2^, ^3^ The analysis of intact proteins by ambient mass spectrometry has been dominated by techniques based on liquid junction surface sampling.^4^ These include liquid extraction surface analysis (LESA),^5^ continuous flow liquid microjunction sampling (commercialised as the Flowprobe),^6^, ^7^ and nano desorption electrospray ionisation (nanoDESI).^8^ Despite its name, nanoDESI is quite different in its fundamental mechanisms to desorption electrospray ionisation (DESI).^9^ DESI is perhaps the most well‐established ambient mass spectrometry technique but until very recently has only been applied to the analysis of small molecules. Exciting results showing DESI of intact proteins from tissue are just starting to emerge.^10^ Figure 1 shows schematics of the ambient mass spectrometry techniques used in the in situ analysis of intact proteins.

It is not possible to describe in situ analysis of intact proteins without mentioning matrix‐assisted laser desorption ionisation (MALDI). MALDI is an ionisation technique which was developed at a similar time as ESI.^11^ It tends to produce singly charged ions, making it most suitable for use with time‐of‐flight instruments which can handle the necessary, extended mass‐to‐charge ranges for the detection of higher molecular weight analytes. MALDI was shown to enable the ionisation and analysis of macromolecules in excess of 10 kDa shortly after its introduction^12^ and has since been successfully used for the analysis of proteins in many contexts. It does, however, have requirements for sample preparation, and analysis is undertaken in vacuum, rendering it significantly different to the ESI‐based ambient ionisation techniques described further in this feature. For reviews describing the applications of MALDI for protein analysis, please see the following.^13^, ^14^, ^15^

### 2.1 Liquid junction surface sampling

As mentioned above, liquid extraction surface sampling encompasses three main techniques: LESA, Flowprobe, and nanoDESI. Each of these has been shown to be suitable for in situ analysis of intact proteins.

#### 2.1.1 Liquid extraction surface analysis

Liquid extraction surface analysis (Figure 1A) is a liquid extraction‐based sampling method coupled to nanoelectrospray ionisation. First described in its current form in 2010 by Kertesz and Van Berkel,^5^ it is most commonly implemented by the use of a TriVersa NanoMate robotic pipette system (Advion, Ithaca, NY). A droplet of solvent is deposited on the sample surface by the electroconductive pipette and held in place to allow the diffusion of analytes into the droplet. This is either achieved via the formation of a liquid junction between the pipette tip and the surface (the standard sampling protocol) or by bringing the pipette tip into contact with the sample surface (contact LESA).^16^ The solvent is then withdrawn and introduced into the mass spectrometer by engaging the tip of the electroconductive pipette with the chip containing a bank of 400 individual nanoelectrospray nozzles. As both the pipette tips and nanoelectrospray nozzles are only used once, there is no possibility of sample carryover.

Liquid extraction surface analysis allows the extraction of all classes of analytes, from small molecules^17^, ^18^, ^19^ up to denatured^16^, ^20^, ^21^ or native‐like proteins and protein complexes,^22^, ^23^ depending on the solvent system used. It is amenable to the analysis of massive intact protein assemblies as demonstrated by detection of the tetradecameric, 800 kDa complex of GroEL (purified protein standard) spotted onto glass slides;^23^ intact haemoglobin tetramers (approximately 64 kDa) have been observed directly in blood spots^22^ and tissue.^24^ High sensitivity is achieved by retaining all extracted analytes in a single droplet of solvent, offering a unique advantage over continuous flow systems. Furthermore, LESA allows great flexibility in experimental design as the sampling and ionisation steps are independent: for example, additional sample manipulation, such as digestion, separation (by ion mobility spectrometry or liquid chromatography), or implementation of multiple consecutive extraction steps (using the same solvent system or multiple solvent compositions).^20^, ^25^, ^26^, ^27^, ^28^, ^29^ These advantages, however, come at the price of the lowest spatial resolution among the ambient ionisation techniques described here. Optical imaging of tissue post‐LESA sampling revealed that for an extraction droplet volume of 0.5 μL dispensed from a height of 0.2 mm, the diameter of the sampled area was ~1158 μm.^30^ That was reduced to ~690 μm, ie, a sampling area of 0.4 mm^2^, by the use of contact LESA^16^ (in which the pipette tip is brought into contact with the tissue). The assumption made is that during contact LESA, the extraction solvent is contained entirely within the pipette tip, which has an internal diameter of ~400 μm. The difference between that value and the measured diameter of the sampling area suggests that some solvent spreading occurs either during extraction or during the raising of the pipette tip following sampling. A perhaps more pressing, and surmountable, issue is that the current commercial software which drives the Triversa Nanomate robot sets a lower limit of 1‐mm spacing between sampling locations.

#### 2.1.2 Flowprobe

The liquid microjunction surface sampling probe, first used for the analysis of TLC plates^31^ and since commercialised as the Flowprobe system (Prosolia), is similar to LESA in that it also relies on the formation of a solvent junction on the sample surface (see Figure 1B). Contrary to LESA, however, it is a continuous flow system. The probe itself consists of two coaxial tubes, measuring approximately 600 μm in diameter. The outer tube contains solvent flowing down towards the sample surface (typically between 10 and 60 μL/min); the inner capillary withdraws it and delivers to a pneumatically aided sprayer attachment for electrospray ionisation. The two flow rates are controlled independently to adjust the size and depth of the liquid junction at the tip of the two tubes.

The Flowprobe offers higher spatial resolution than the LESA apparatus, reliably achieving a sampling area diameter of ~600 μm (limited by the dimensions of the probe tip, similarly to LESA). There are two modes of operation: “spot mode” (also known as “array mode”) in which the probe is held at a single location before it is raised (and flushed) before sampling the next location and “raster mode” in which the sample stage is moved (rastered) under the probe at a constant speed, maintaining a liquid junction. The probe is flushed at the end of each raster line, and 1 data file corresponds to a single raster line. Raster mode sampling allows the reduction of pixel sizes to a minimum of 50 μm in the *x*‐dimension; however, that is not recommended because of oversampling effects.^32^ The sensitivity of the system is much lower than that of LESA, both as a factor of the smaller sampling area as well as the continuous flow design which dilutes extracted analytes.^32^, ^33^ It does, however, provide a higher extraction efficiency as a liquid junction can be maintained for extended periods of time in a single location and continuously extracted with fresh solvent.^33^ Because of the similarity in the principle of operation, these effects are also shared with nanoDESI, described in more detail below. Unlike nanoDESI, however, this design is not self‐aspirating, and therefore, the balancing of the flow rates towards (between 10 and 60 μL/min) and away (controlled by gas pressure up to 100 psi) from the sample surface to achieve the desired size of the liquid junction requires careful adjustment.^7^ The dynamics of the fluid in the liquid junction itself affect extraction efficiency, introducing additional variability into the system; this can be partially controlled by altering the geometry of the probe.^34^ As an additional, practical consideration, the acetonitrile‐based solvent systems which provide optimal protein extraction are difficult to use with the commercially available Flowprobe platform because of the polyimide coating of the capillaries, which swells upon exposure to acetonitrile.^32^, ^35^ Nevertheless, proteins with molecular weights up to 15 kDa have been detected from tissue.^32^, ^36^

#### 2.1.3 NanoDESI

Nano desorption electrospray ionisation is an alternative continuous flow, liquid junction‐based technique (Figure 1C).^8^ The apparatus consists of two capillaries held at an angle with respect to each other, with a small gap left between the capillaries at the sampling surface. Solvent is continuously fed through the first capillary, at the end of which it spills onto the sample in a controlled manner, forming the liquid junction. It is aspirated into the second capillary and expelled from the other end in a nanoelectrospray mist towards the inlet of the mass spectrometer. The careful adjustment of the two capillaries to reduce the size of the liquid junction still delivers the best spatial resolution of all liquid sampling‐based techniques, estimated at 10 μm as determined by sampling a rhodamine standard grid.^37^ Pixel diameters of 20 μm or better were achieved on tissue sections^37^, ^38^ although similar results are not trivial to achieve on uneven surfaces; this is because of the extremely small distance between the capillaries and the sample surface (roughly equal to the desired pixel diameter) which needs to be reliably established and maintained over the course of an imaging experiment. The basic nanoDESI set‐up cannot achieve such precise control on samples of variable height, and thus, the size of the liquid bridge needs to be increased to absorb the differences without leading to loss of signal either through collision of the apparatus with the sample or loss of contact between the sample and the liquid bridge; the spot diameter could thus increase to approximately 1 mm for very rough samples such as bacterial colonies.^39^ This limitation was recently overcome by the integration of a shear force probe alongside the nanoDESI probe.^40^, ^41^ Thus, the topography of the sample can be measured and fed back to the control interface of the apparatus during a raster scan across the sample surface, allowing for the continuous adjustment of the probe positioning to maintain optimum distance from the sample. Whilst the majority of nanoDESI work has focused on metabolites and lipids, proteins up to 15 kDa have been imaged in thin tissue sections.^42^

### 2.2 DESI

Unlike the previously mentioned techniques, DESI (Figure 1D) does not involve the formation of a liquid junction.^9^ Sampling is achieved by directing a jet of charged solvent at the sample surface; charged particles of solvent impact the surface, desorbing molecules of analyte and imparting electrical charge. Analyte ions are then picked up by a transfer tube attached directly to the inlet of the mass spectrometer and delivered for analysis. DESI requires a hard, nonconductive surface to yield optimal results. The technique was initially suitable only for the reliable analysis of purified and relatively small (<25 kDa) proteins under denaturing conditions^43^, ^44^, ^45^; this was shown to be a consequence of undesirable protein‐protein or protein‐contaminant clustering and incomplete dissolution of the analytes.^44^ Progress has since been made to mitigate these effects by use of solvent additives.^46^ In another approach, by modifying the DESI set‐up itself, DESI mass spectra of native protein complexes of up to 800 kDa in size (tetradecameric GroEL) spotted onto glass slides have recently been recorded.^47^ An alternative approach for analysing the surface layer of liquid samples generated ions of protein complexes of approximately 150 kDa.^48^ Although intact purified proteins spotted onto glass slides have been observed via DESI, to date the detection of intact protein species directly from thin tissue sections has proved challenging. Very recently, Towers et al^10^ have shown that by modifying the DESI source and incorporating ion mobility spectrometry, it is possible to detect proteins from tissue.

## 3 TOP‐DOWN IDENTIFICATION OF PROTEINS

A key step in the in situ analysis of proteins is their identification. Identification involves fragmentation (tandem mass spectrometry (MS/MS)) of the intact protein ion. The resulting fragment ions provide information on the primary sequence of the protein. The measured mass‐to‐charge (m/z) ratios of the fragment ions are searched against theoretical m/z values generated from a protein database, thereby enabling protein identification. This process is known as top‐down mass spectrometry.^49^

**Figure 2:**
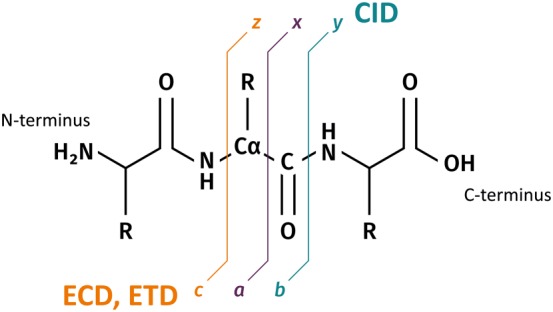
Peptide fragmentation nomenclature.^50^ *b*, *y*, and a minor proportion of *a*‐type fragments are produced by collision‐induced dissociation; electron capture and electron transfer dissociation generate chiefly *c* and *z*‐type ions, with some *a* and *y* ions

Top‐down protein analysis can be achieved via a range of fragmentation techniques, most commonly collision‐induced dissociation (CID), electron capture dissociation (ECD), or electron transfer dissociation (ETD). CID involves acceleration of analyte ions into particles of an inert gas, such as helium. Inelastic collisions between the two result in conversion of some of the kinetic energy of the ion into internal vibrational energy and subsequent bond cleavage via the lowest energy pathways. For peptides and proteins, CID results in cleavage of the amide bonds to produce *b* and *y* fragments^50^; see Figure 2. (Notably, a small proportion of *a*‐type fragments can also be observed in CID mass spectra.) CID is perhaps the most widely used method of fragmentation, available on a range of instruments, although it does not yield the highest sequence coverage, particularly when disulfide bonds are present.

Electron capture dissociation has shown significant promise in the fragmentation of larger analytes.^51^, ^52^ ECD of peptides and proteins involves the irradiation of multiply‐charged precursor cations with low‐energy electrons, resulting in capture of an electron and cleavage of the N─Cα bond to produce *c* and *z* fragment ions (see Figure 2).^53^, ^54^ Because the addition of an electron reduces the charge of the ion, this type of fragmentation is only suitable for ions of charge state 2+ and higher. An advantage of ECD for top‐down mass spectrometry is that cleavage is random, and therefore, sequence coverage tends to be higher. Moreover, labile modifications are retained on the backbone fragments. It is particularly useful for identifying the precise location of putative posttranslational modifications as well as de novo protein sequencing.^55^, ^56^

Electron transfer dissociation was introduced in 2004^57^ and is closely related to ECD. ETD is a two‐step process involving transfer of an electron from a radical anion (most commonly fluoranthene) to a protein precursor ion.^54^ Fragmentation thus relies on very similar principles as ECD and yields the same fragment types (mainly *c* and *z*) (see Figure 2). Despite its later introduction, ETD has been applied to top‐down protein analysis much more frequently than ECD as it is available in a greater range of mass analysers, including orbitrap and time‐of‐flight instruments; by contrast, ECD is largely confined to FT‐ICR mass spectrometers.^54^ A distinct advantage of ETD over CID is its tendency to preserve labile posttranslational modifications, which allows for their identification and localisation.^58^

Irrespective of the method by which they were obtained, fragmentation mass spectra may be used to identify the original protein precursor. Whilst it is theoretically possible to generate de novo a partial or even complete amino acid sequence based on the fragmentation data, the efficiency of bond cleavage is frequently too low to make this a viable approach. Instead, dedicated protein database search algorithms have been developed which take into account the mass of the intact precursor, the masses of fragments generated from the tandem mass spectrum, or both, to return a list of putative identifications. ProSight PTM,^59^ later developed into ProSightPC (Thermo Fisher Scientific), and MS‐Align+^60^ are most commonly used; both algorithms rely on selecting putative candidate sequences from a protein database, based on the intact mass of the precursor, and subsequently matching observed fragment masses against a list of theoretical fragment masses generated from the database. Both also provide a scoring mechanism for the statistical evaluation of protein‐spectrum matches.

As briefly discussed above, the top‐down analysis of intact proteins offers unique advantages over the commonly used bottom‐up methodology involving enzymatic digestion of extracted proteins prior to their analysis. The most immediate boon is the rapid analysis time, within the range of a few minutes, compared to the slow turnover of LC/MS (approximately 1 hour per sample). The provision of an accurate intact mass allows the prediction of single amino acid substitutions and functionally relevant posttranslational modifications which may not be detected on the small subset of observable peptides generated by enzymatic cleavage from each individual protein. Furthermore, the folded structure of the observed proteins can be retained by use of gentle native‐like sampling conditions, enabling a degree of structural characterisation of protein complexes and the study of noncovalent interactions. Whilst these features render top‐down protein mass spectrometry extremely powerful for the analysis of specific proteins or subsets of proteins from biologically relevant samples, they currently come at the price of a severely reduced depth of protein coverage as compared to bottom‐up proteomics. This is because of the complexity of the mixture of very large analytes each exhibiting different physicochemical properties and occupying a wide range of charge states, the great dynamic range of the proteome still exceeding that of modern instrumentation, as well as the vast number of isoforms and multiple dynamic modifications which need to be considered for successful identification, all compounded by the difficulty in generating high‐quality MS/MS data from larger proteins.^61^ The separation step integral to proteomics is frequently omitted in the case of the ambient ionisation techniques described above, further reducing the breadth of detected proteins in exchange for a rapid analysis time. Thus, the top‐down and bottom‐up approaches are currently complementary rather than competitive.

## 4 AMBIENT IN SITU ANALYSIS OF INTACT PROTEINS IN BIOLOGICAL SUBSTRATES

### 4.1 Dried blood spots

Liquid extraction surface analysis mass spectrometry was first described for the analysis of intact proteins from dried blood spots (DBS) in 2011.^62^ Haemoglobin comprises two α‐globin and two β‐globin polypeptide chains, each noncovalently bound to prosthetic heme groups. LESA sampling of DBS using aqueous organic solvent systems results in detection of α‐globin and β‐globin ions in a range of charge states. Variants of haemoglobin are the most commonly inherited disease, with over >1700 variants known.^63^ Variants are either because of point mutations in a globin gene resulting in a single amino acid substitution in a globin chain (with an associated shift in mass) or a reduction in synthesis of one of the globin chains (known as thalassemias). Initial experiments revealed that top‐down LESA mass spectrometry could be used to diagnose the variants HbS (sickle; E6V, Δ*m* 29.97 Da), HbC (E6K, Δ*m* 0.95 Da), and HbD (E121Q; Δ*m* 0.98 Da) in DBS from newborns.^62^ Subsequent work showed the approach could be applied to the diagnosis of HbE (E26K; Δ*m* 0.95 Da), HbD‐Iran (E22Q; Δ*m* 0.98 Da), Hb Headington (S72R; Δ*m* 69.1 Da), Hb J Baltimore (G16D; Δ*m* 58.0 Da), and Hb Phnom Penh (insertion of isoleucine between amino acids 117 and 118 on the α‐globin; Δ*m* 113.1 Da), as well as detecting the presence of β thalassemia major, in newborn DBS.^64^, ^65^

As mentioned above, an advantage of LESA is the flexibility to decouple the sampling and ionisation steps. This flexibility was exploited in a LESA proteomic analysis of DBS in which intact proteins were extracted via LESA and then digested with trypsin prior to liquid chromatography tandem mass spectrometry (LC‐MS/MS). Using this approach, 120 proteins were identified from a single DBS over a concentration range of 4 orders of magnitude, highlighting the sensitivity of this approach.^25^

Direct sampling of proteins from DBS has primarily been undertaken by LESA. Nevertheless, an early version of the Flowprobe was also applied to the analysis of DBS from sheep. The continuous‐flow solvent sampling probe was coupled with liquid chromatography, enabling the detection of the α‐globins and β‐globins.^33^

### 4.2 Thin tissue sections

Although LESA mass spectrometry has been widely applied to the analysis of small molecules (eg, drugs and their metabolites),^5^, ^66^, ^67^, ^68^, ^69^, ^70^ comparatively few reports describe intact protein analysis from tissue. Schey et al^71^ applied manual LESA extraction to sections of bovine ocular lens and mouse brain and kidney. LESA sampling was followed by liquid chromatography top‐down ETD MS/MS of the extracted proteins. Intact and truncated crystallins (MW ~20‐22 kDa) were identified in the lens sample, and a range of proteins in the molecular weight range 4 to 22 kDa were identified in the brain and kidney samples.

Automated LESA top‐down mass spectrometry of tissue was first demonstrated for the analysis of intact proteins in human nonalcoholic steatohepatitis (NASH) tissue.^26^ Liver fatty acid binding protein (FABP1) and its variant (T94A) (a putative biomarker of NASH^72^) were identified by ETD and CID MS/MS, in addition to the 10 kDa heat shock protein and α‐globin.

**Figure 3:**
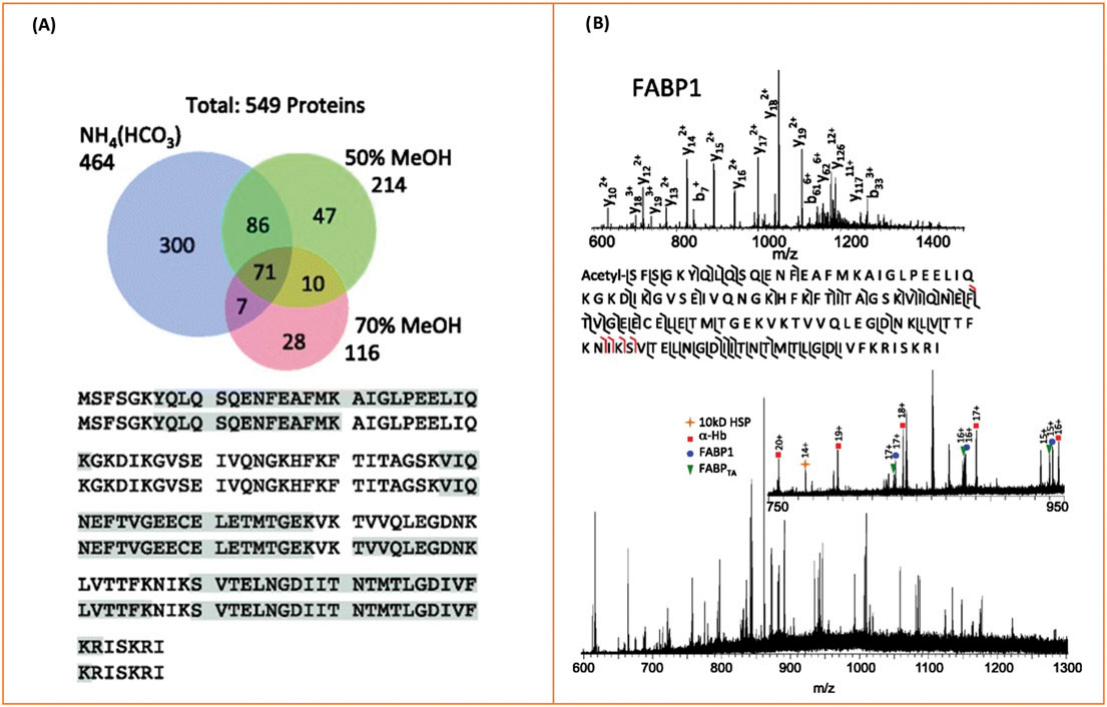
Liquid extraction surface analysis MS of human nonalcoholic steatohepatitis tissue. A, Liquid extraction surface analysis followed by bottom‐up LC MS/MS analysis: Numbers of proteins identified following extraction by use of three different solvents and the sequence coverage obtained for fatty acid binding protein (FABP1) extracted in ammonium bicarbonate. B, Top‐down CID spectrum and sequence coverage showing protein identification of FABP1; further identified proteins are marked in the full‐scan mass spectrum below. Adapted and reproduced from J. Sarsby, N. J. Martin, P. F. Lalor, J. Bunch and H. J. Cooper, Journal of the American Society for Mass Spectrometry, 2014, 25 (11) p 1953‐1961. DOI: 10.1007/s13361‐014‐0967‐z. Published by Springer US under the terms of the Creative Commons Attribution Licence (http://creativecommons.org/licenses/by/4.0/)

That work also used a “bottom‐up” approach in which intact proteins were extracted via LESA and subsequently digested by trypsin prior to analysis of the resulting peptides by LC‐MS/MS (Figure 3). Over 500 proteins were identified; however, the FABP1 variant was not reproducibly identified via the bottom‐up approach. These results emphasise that whilst bottom‐up approaches provide broad proteome coverage, a top‐down approach is more suitable for comprehensive analysis of individual proteins by enabling identification and localisation of single amino acid substitutions. Similar bottom‐up approaches have been applied to DBS^25^ (see details above) and formalin‐fixed paraffin‐embedded ovarian cancer tissue.^73^ Wisztorski et al have applied a bottom‐up approach for spatially directed extraction of intact proteins from thin tissue sections of mouse brain, prior to digestion and analysis of the resulting peptides by LC‐MS/MS.^74^ Over 1400 proteins were identified from a 1‐mm pixel location.

Recently, Lamont et al^27^ replaced the conventional pipette tip used in LESA with a silica capillary (typically used for coupling with LC fraction collection) thus reducing the sampling area to 400 μm diameter. LESA sampling of rat pituitary was coupled with LC and data‐independent MS/MS (MS^E^). The majority of species identified were peptides up to ~6 kDa; however, the 20 kDa protein proopiomelanocortin was also identified.

**Figure 4:**
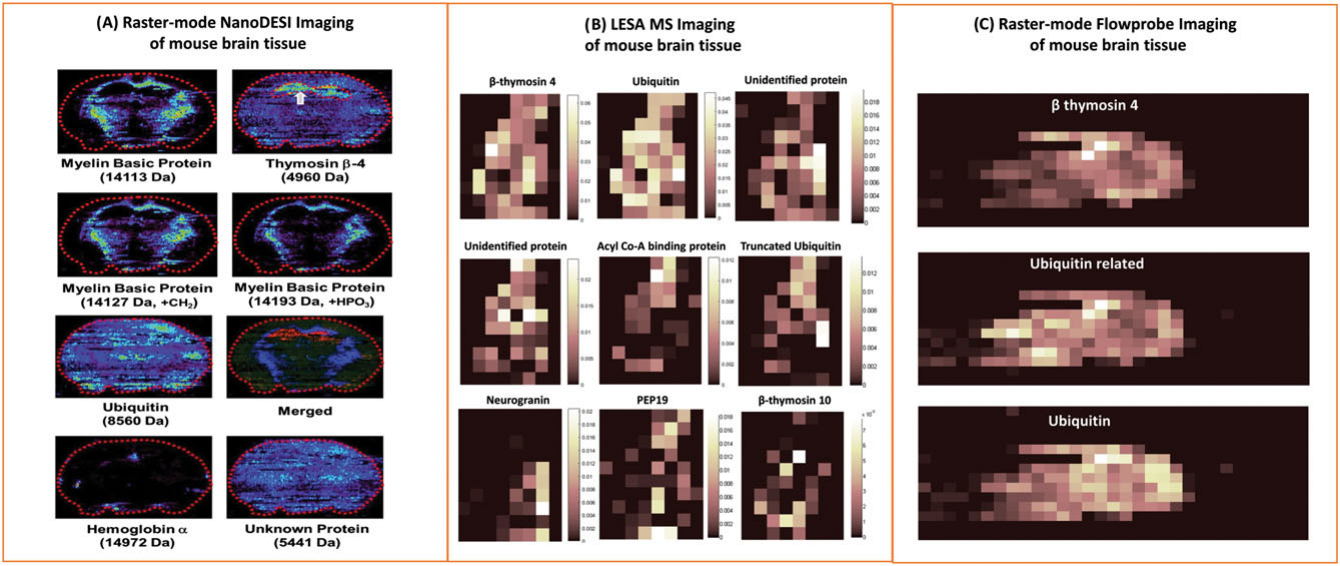
Ambient mass spectrometry imaging of intact proteins from mouse brain tissue via A, nano desorption electrospray ionisation, B, liquid extraction surface analysis, and C, raster‐mode Flowprobe. Adapted and reproduced with permission from A, C. Hsu, P. Chou and R. N. Zare, Analytical Chemistry, 2015, 87 (22); p 11171‐11175. DOI: 10.1021/acs. analchem. 5b03389, Copyright 2015 American Chemical Society, B, R. L. Griffiths, E. K. Sisley, A. F. Lopez‐Clavijo, A. L. Simmonds, I. B. Styles and H. J. Cooper, International Journal of Mass Spectrometry, 2017, *In Press*. DOI: 10.1016/j.ijms. 2017.10.009. Published by Elsevier under the Creative Commons Attribution Licence (CC‐BY) (http://creativecommons.org/licenses/by/4.0/), and C, R. L. Griffiths, E. C. Randall, A. M. Race, J. Bunch and H. J. Cooper, Analytical Chemistry, 2017, 89 (11); p 5683‐5687. DOI: 10.1021/acs. analchem. 7b00977. Published under the Creative Commons Attribution Licence (CC‐BY). Published 2017 by American Chemical Society

A major application of ambient in situ analysis of thin tissue sections is mass spectrometry imaging. By sampling in a sequential grid‐like fashion, an array of mass spectra, each associated with a particular location, is amassed. From these data, ion images can be generated, showing the spatial distribution of different analytes. NanoDESI mass spectrometry imaging has been applied to coronal sections of mouse brain^42^: Ubiquitin, β‐thymosin 4, α‐globin, and myelin basic proteins (ie, up to ~15 kDa) were identified and spatially mapped; see Figure 4A. The approach was also applied to healthy and lymphoma thymus tissue. The protein β‐thymosin 10 was additionally identified in the thymus tissue, and the results showed increased truncation (for proteins ubiquitin, β‐thymosin 4, and β‐thymosin 10) in the diseased tissue. The spatial resolution achieved by nanoDESI was ~200 μm.

Liquid extraction surface analysis MS imaging of mouse liver and brain tissue has also been described^29^, ^75^; 15 and 24 intact protein species were detected across thin tissue sections of brain and liver respectively in the range up to 16 kDa.^29^ That study also demonstrated the benefits of incorporating ion mobility separation into imaging workflows; 34 proteins (26 unique) and 40 proteins (29 unique) were detected from mouse brain and liver respectively when high field asymmetric ion mobility spectrometry (FAIMS) was included in the workflow. This aspect is discussed in more detail below. LESA MS imaging by the use of native‐like solvents of intact proteins up to 15 kDa in mouse brain has also been demonstrated^75^; see Figure 4B.

More recently, Flowprobe mass spectrometry of intact proteins from thin tissue sections has been demonstrated both in the presence (see below for further discussion) and absence of ion mobility spectrometry.^32^, ^36^ The latter study involved collection of data in raster mode, ie, the sample stage was continuously moved beneath the sample probe, from sections of mouse brain. The results revealed rapid ambient surface sampling analysis of intact proteins, providing significant time benefits over spot‐mode Flowprobe sampling and LESA approaches. Imaging data acquisition for a sagittal mouse brain tissue section at 600 μm resolution took ~1 hour via Flowprobe MS in raster imaging mode, whereas imaging of an equivalent area 600 μm array in spot mode would take ~10 hours. Nevertheless, improved throughput comes with a compromise in sensitivity for intact proteins (in the absence of ion mobility separation); fifteen intact protein species were reported via LESA MS imaging of mouse brain,^29^ whereas only three intact protein species are described in similar Flowprobe experiments; see Figure 4C.^32^ Moreover, whilst pixel sizes of 50 μm are achievable, the optimum spatial resolution is ~600 μm to avoid oversampling artefacts. That is, the spatial resolution remains the same as spot‐mode Flowprobe sampling (~600 μm) which is similar to the internal diameter of the LESA pipette tip (~400 μm), which is the best achievable resolution with LESA.

### 4.3 Microorganisms

The study of microbial proteins derived directly from living colonies presents an inherent challenge because of the requirement for cell lysis prior to or during sampling. Initial in situ studies of microorganisms by ambient ionisation techniques focused on intra‐species and inter‐species interactions observed between colonies grown on agar media, as well as the characterisation of the microbes' metabolic output. Whilst the majority of the techniques used only supplied data on small molecules, two liquid extraction‐based methodologies, nanoDESI and the Flowprobe, also revealed the presence of small (up to 4.5 kDa) secreted peptides^39^, ^76^ in and around the colonies of selected bacterial strains.

The properties of bacterial colonies challenge each of these techniques in unique ways. NanoDESI struggles particularly with the variability in sample height^39^; any accidental contact with the colony risks obstructing solvent flow through the system, interrupting the nanospray and necessitating cleaning or exchange of the capillaries. The Flowprobe is less susceptible to such issues because of the larger diameters of the capillaries used, although contact with the colony is still undesirable as it introduces contamination into the system, as well as increasing the risk of sample carryover.^76^ Neither technique is, however, currently capable of extracting cytosolic proteins.

**Figure 5:**
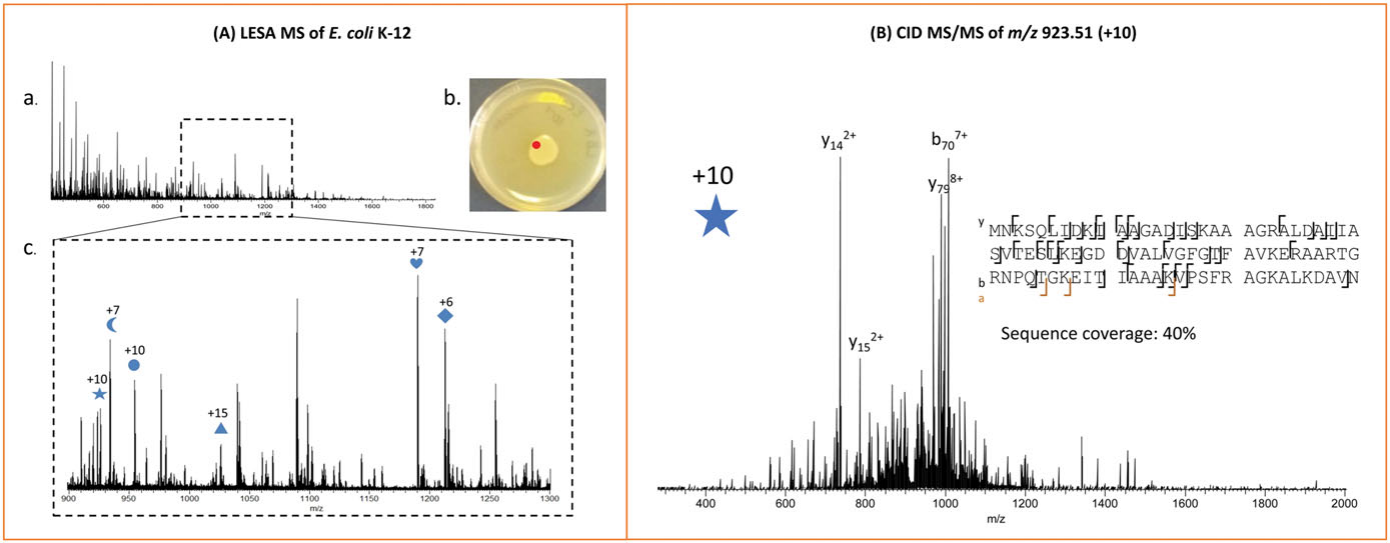
Liquid extraction surface analysis mass spectrometry of E. coli K‐12. A, Representative full‐scan mass spectrum of a colony stored at 4°C, sampled at the location marked in red. The m/z region containing most of the observed protein peaks is shown below. B, CID mass spectrum of ions centred at m/z 923.51, charge state +10 (marked with a star in the full‐scan mass spectrum). The protein was identified as the DNA‐binding protein HU‐β. Adapted and reproduced from E. C. Randall, J. Bunch, and H. J. Cooper, Analytical Chemistry, 2014, 10504‐10510. DOI: 10.1021/ac503349d. Published under the Creative Commons Attribution Licence (CC‐BY). Published 2014 by American Chemical Society

**Figure 6:**
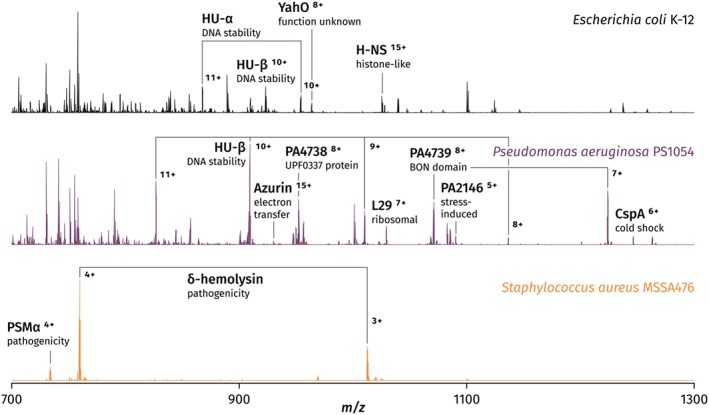
Contact liquid extraction surface analysis mass spectra of three representative bacterial species: Escherichia coli K‐12, *Pseudomonas aeruginosa* PS1054, and Staphylococcus aureus MSSA476. Adapted and reproduced from K. I. Kocurek, L. stones, J. Bunch, R. C. May, and H. J. Cooper, Journal of the American Society for Mass Spectrometry, 2017, 28 (10); p 2066‐2077. DOI: 10.1007/s13361‐017‐1718‐8. Published by Springer US under the Creative Commons Attribution 4.0 International Licence (http://creativecommons.org/licenses/by/4.0/)

Liquid extraction surface analysis mass spectrometry was the first technique successfully used for the extraction of periplasmic and cytosolic proteins directly from living bacterial colonies.^16^ The initial results were demonstrated on E. coli K‐12, a model laboratory strain (Figure 5). Six proteins were identified by CID followed by matching deconvoluted fragmentation spectra against an E. coli protein database included in the ProSightPTM 2.0 software (freely available online). Crucially, the observation of cytosolic proteins was possible only by driving the extraction pipette tip into contact with the colony, most likely inducing mechanical lysis of bacterial cells. Neither nanoDESI nor the Flowprobe are capable of replicating this manoeuvre because of issues with capillary clogging described above; whilst LESA nanoelectrospray can also be hindered by intake of colony material into the pipette tip, the large diameter as well as the single‐use nature of the pipette tips greatly reduces the severity of such issues. Following this proof of concept, further work was carried out on both E. coli K‐12 and E. coli BL21, and a range of clinical isolates, including gram‐negative *Pseudomonas aeruginosa* PS1054, gram‐positive Staphylococcus aureus MSSA476, and three closely related species of streptococci^21^ (see Figure 6). It was found that the LESA solvent comprising 40:60:1 acetonitrile/water/formic acid developed for sampling of E. coli was not suitable for the peptidoglycan‐rich cell walls of gram‐positive species; a new extraction solvent, with an increased content of acetonitrile (50%) and formic acid (5%), was optimised for this purpose. A subset of over 40 proteins from the total observed in the 7 species, with molecular weights ranging between 3 and 15 kDa, were selected for CID followed by automatic fragment matching using ProSightPC 3.0 software (Thermo Fisher Scientific). Multiple protein types, deriving from colony surfaces, the periplasm, and the cytoplasm, were observed, including ribosomal, DNA‐binding, and membrane‐binding proteins. A large variety of stress response factors were also observed, in particular the UPF0337 family, members of which were observed in all sampled species. The identification and de novo sequencing of a novel protein detected in an unidentified species of *Staphylococcus* was also demonstrated. The CID data obtained by LESA mass spectrometry allowed for the reconstruction of a nearly complete sequence subsequently fed into a homology search, which returned no matches. Thus, it was shown that LESA mass spectrometry could potentially be used for the identification of novel proteins and peptides without the need for pre‐existing genomic data.

The number of proteins detected by LESA mass spectrometry, using E. coli as the example, was an order of magnitude lower than that obtained by top‐down LC/MS^77^ or bottom‐up LC/MS of bacterial culture extracts (over 150 and 300‐450 respectively)^78^, ^79^; the analysis time by LESA mass spectrometry is, however, significantly lower than it is for the above techniques (less than 5 minutes versus a minimum of 1 hour), demonstrating a significant time benefit. This, combined with the lack of sample preparation, makes LESA mass spectrometry particularly useful for rapid phenotypic screening. Whilst MALDI mass spectrometry has also been used for similar purposes,^80^ it is unsuitable for the analysis of living microbes directly on media. Moreover, it was demonstrated that LESA mass spectrometry is capable of differentiating viridans group streptococci on the basis of the differing intact masses of observed proteins. This is a known challenge for MALDI‐TOF‐MS because of the high similarity of fingerprint mass spectra among these particular species.

## 5 INCLUSION OF ION MOBILITY SPECTROMETRY IN AMBIENT MASS SPECTROMETRY WORKFLOWS

A major challenge in the direct sampling of biological substrates is the inherent complexity of the sample. That is, many molecular classes are present (proteins, peptides, lipids, carbohydrates, etc.), and all may be extracted and may interfere with detection of the analyte of interest, in this case proteins. Moreover, proteins may be present over a wide concentration range, with higher abundance proteins masking the presence of lower abundance proteins. One potential approach for addressing these challenges is to incorporate liquid chromatography^27^, ^73^, ^74^; however, a considerable disadvantage of liquid‐phase separation techniques is the time cost. A typical protein or peptide HPLC analysis takes tens of minutes to an hour, making that approach incompatible with mass spectrometry imaging. For example, if HPLC was integrated, it would take a day to collect data for an image comprising just 24 pixels. In contrast, the gas‐phase separation afforded by ion mobility spectrometry can be achieved on the order of milliseconds. To date, two ion mobility spectrometry approaches have been integrated with ambient mass spectrometry of intact proteins: high field asymmetric waveform ion mobility spectrometry (FAIMS, also known as differential ion mobility spectrometry)^81^, ^82^, ^83^ and travelling wave ion mobility spectrometry (TWIMS).^84^

### 5.1 High field asymmetric waveform ion mobility spectrometry

**Figure 7:**
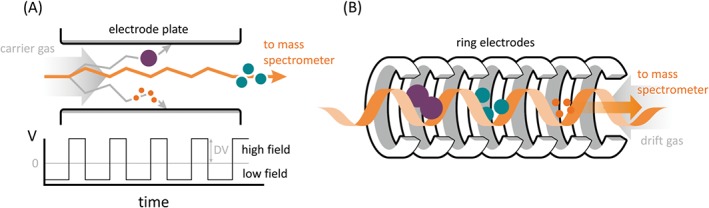
Schematics of ion mobility separation techniques. A, High field asymmetric waveform ion mobility separation. B, Travelling wave ion mobility separation

High field asymmetric waveform ion mobility spectrometry,^82^ also known as differential ion mobility spectrometry, separates gas‐phase ions at atmospheric pressure on the basis of differences in their ion mobilities in high and low electric fields. Ions are transported by a carrier gas between parallel electrodes to which an asymmetric waveform is applied. See Figure 7A. The ions therefore experience alternating high and low electric fields. The high electric field is referred to as the dispersion field, the result of the dispersion voltage. As the ions have different mobilities in the high and low electric fields, they are displaced from their original trajectory through the device and in the absence of intervention will collide with one or other of the electrodes. To prevent this, a compensation field is superposed. By scanning the compensation field, ion with different mobilities are transmitted through the FAIMS electrodes, and in this way, the ion mobility device acts as an ion filter.

The benefits of the incorporation of FAIMS separation into the mass spectrometry workflow have been described for a variety of the ambient techniques described above. The incorporation of FAIMS into the workflow provides molecular separation and reduced chemical noise, both of which increase the range of ions detected with acceptable signal‐to‐noise ratios. LESA FAIMS mass spectrometry has been demonstrated for living bacterial colonies,^20^ thin tissue sections,^20^ and DBS^28^ and has also been described in imaging workflows.^29^ The inclusion of FAIMS in LESA mass spectrometry workflows led to an increase in the number of intact proteins detected. For a single location in mouse brain, the number of intact proteins (5‐37 kDa) detected increased from 3 to 29 following inclusion of FAIMS.^20^ LESA FAIMS mass spectrometry of E. coli growing on agar resulted in identification of the acid stress chaperone protein HdeA which had not been detected in the absence of FAIMS.^20^ Furthermore, the use of the FAIMS device as an ion filter allows separation of molecular classes; hence, lower abundance species can be detected in the presence of other highly abundant species. This advantage was demonstrated nicely for the analysis of lipid species in the presence of haemoglobin protein species from DBS.^28^

**Figure 8:**
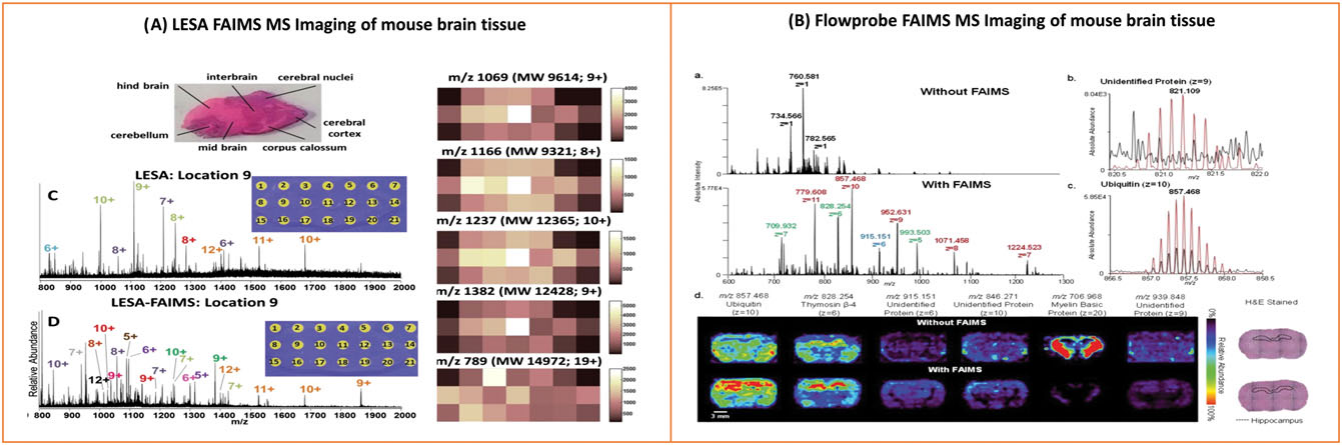
Example mass spectra and ion images of mouse brain tissue demonstrating the benefits of incorporating field asymmetric waveform ion mobility separation into liquid extraction surface analysis and Flowprobe MS workflows. Reproduced from (A) R. L. Griffiths, A. M. Race, A. J. Creese, J. Bunch, and H. J. Cooper, Analytical Chemistry, 2016, 88 (13), p 6758‐6766, DOI: 10.1021/acs. Analchem. 6b01060, published under the Creative Commons Attribution Licence (CC‐BY), published 2016 by American Chemical Society and (B) C. L. Feider, N. Elizondo, and L. S. Eberlin, Analytical Chemistry, 2016, 88 (23) p 11533‐11541, DOI: 10.1021/acs. Analchem. 6b02798. Copyright 2016 American Chemical Society

Incorporating FAIMS into LESA MS imaging workflows led to similar benefits across whole tissue sections^29^; 34 intact proteins, 26 of which were unique to the FAIMS experiment, were reported across a mouse brain tissue section; see Figure 8. Furthermore, 40 intact proteins, 29 unique to the FAIMS experiment, were reported across a mouse liver tissue section.^29^ Similar benefits have been described for Flowprobe MS FAIMS imaging of mouse brain tissue and human ovarian cancer tissue samples; 84 intact proteins, 66 of which were unique to the FAIMS workflow, were reported across a rat brain tissue section.^36^

### 5.2 Travelling wave ion mobility spectrometry

An alternative gas‐phase separation method is TWIMS.^84^ Unlike classical (drift tube) ion mobility spectrometry, which uses a uniform electric field to drive ions through a cell of known length containing a buffer gas, TWIMS makes use of nonuniform transient DC pulses along a stacked‐ring ion guide (producing a “travelling wave”) to drive ions through the buffer gas; see Figure 7B. By reducing the height of the travelling wave and increasing the pressure in the device, some ions will roll over the wave, thereby increasing their transit time. Lower mobility ions experience more rollover events than higher mobility ions, enabling ion mobility‐based separation. The trajectory of the ions through the travelling wave device is complex: To determine collision cross sections (CCS) for the ions, it is necessary to calibrate with species of known CCS measured from drift tube ion mobility measurements. Nevertheless, unlike FAIMS, calculation of CCS is possible via TWIMS.

A number of ambient surface sampling techniques have been coupled with TWIMS. Lamont et al^27^ coupled LESA with liquid chromatography and TWIMS for the separation of isobaric peptide hormones extracted from rat brain. The separation afforded by TWIMS proved vital in the detection of intact proteins from thin tissue sections via DESI.^10^ LESA coupled with TWIMS has been applied to the measurement of CCS of folded proteins extracted from thin tissue sections of mouse brain^75^ (see below). It is also worth noting that (classical) drift tube ion mobility spectrometry has been coupled with DESI for the investigation of gas‐phase structures of pure cytochrome c and lysozyme.^45^

## 6 NATIVE LESA MS

**Figure 9:**
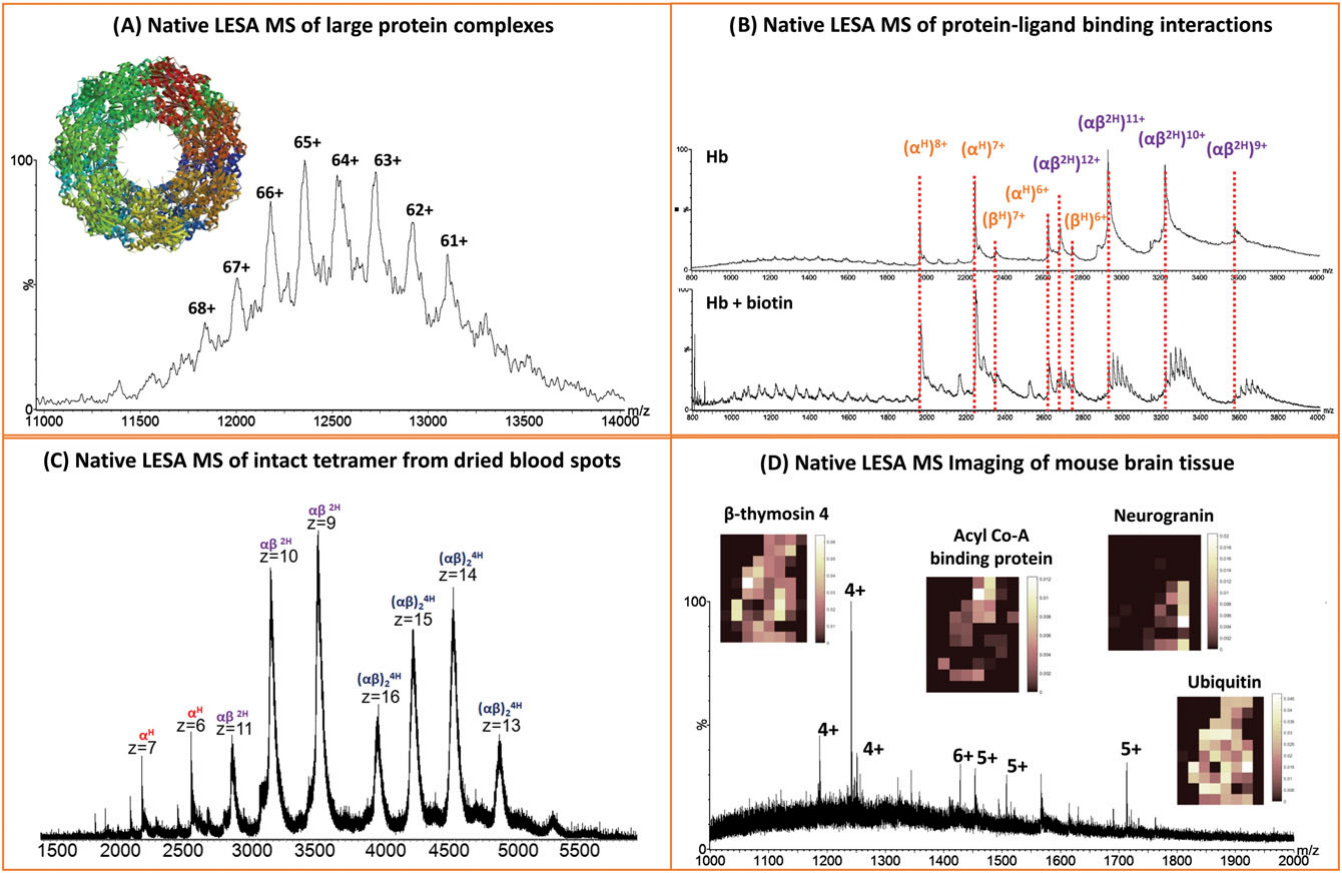
Native liquid extraction surface analysis (LESA) MS. A, Native LESA MS of tetradecameric GroEL (~800 kDa). B, Native LESA MS of biotin binding to haemoglobin. C, Native LESA MS of tetrameric haemoglobin extracted from dried blood spots. D, Native LESA MS imaging of mouse brain tissue with selected ion images. Adapted and reproduced from (A) and (B) V.A. Mikhailov, R. L. Griffiths, and H. J. Cooper, International Journal of Mass Spectrometry, 2017, (420), 43‐50. DOI: 10.1016/j.ijms. 2016.09.011. Published by Elsevier under the Creative Commons Attribution Licence (CC‐BY) (http://creativecommons.org/licenses/by/4.0/), (C) N. J. Martin, R. L. Griffiths, R. L. Edwards, and H. J. Cooper, Journal of the American Society for Mass Spectrometry, 2015, (8), 1320‐7. DOI: 10.1007/s13361‐015‐1152‐8. Published by Springer US under the Creative Commons Attribution 4.0 International Licence (http://creativecommons.org/licenses/by/4.0/), and (D) R. L. Griffiths, E. K. Sisley, A. F. Lopez‐Clavijo, A. L. Simmonds, I. B. Styles, and H. J. Cooper, International Journal of Mass Spectrometry, 2017, *In Press*. DOI: 10.1016/j.ijms. 2017.10.009. Published by Elsevier under the Creative Commons Attribution Licence (CC‐BY) (http://creativecommons.org/licenses/by/4.0/)

Native mass spectrometry is a burgeoning field in which, using carefully selected buffer solutions, weak noncovalent interactions such as hydrogen bonding and salt bridges are maintained during electrospray ionisation. This capability enables gas‐phase analysis of macromolecular structures, reviewed in Mehmood et al.^85^ Recently, similar ammonium acetate‐based solvents have been implemented as LESA^22^, ^23^, ^24^, ^75^ extraction solvents for the study of native‐like intact proteins and protein complexes directly from solid substrates. Native LESA mass spectrometry of purified protein assemblies dried onto glass substrates has been demonstrated.^23^ Tetrameric avidin (~64 kDa), octameric (~190 kDa) and hexadecameric (~380 kDa) CS_2_ hydrolase, and tetradecameric GroEL (~800 kDa) (see Figure 9A) were all detected. In addition, the trimeric membrane protein AmtB (~140 kDa), dried onto the substrate from a solution containing C8E4 micelles, was detected intact following LESA using a native‐like solvent containing micelles. Native LESA mass spectrometry was also shown to be suitable for probing protein ligand‐binding interactions. Noncovalent complexes between the ligand biotin and proteins avidin, bovine serum albumin, and haemoglobin were detected as shown in Figure 9B. Similar studies have recently been described for native DESI analysis of purified samples of intact proteins and protein assemblies; Ambrose et al describe detection of monomeric proteins such as apo lysozyme and bovine serum albumin, complexes of tetrameric alcohol dehydrogenase and tetradecameric GroEL using ammonium acetate solutions.^47^ They also show that native DESI is suitable for the analysis of membrane proteins, although some detergent sensitivity is exhibited, and for probing noncovalent protein interactions in the example of NAG‐5 bound to lysozyme.

The work on native LESA MS of purified protein assemblies and protein‐ligand complexes followed earlier work in which it was demonstrated that the haemoglobin tetramer complex ((α^H^β^H^)_2_) could be detected directly from DBS^22^ (Figure 9C) and vasculature present within tissue sections.^24^ In that work, contact‐LESA sampling (described earlier) proved particularly beneficial for improving native protein signal. More recently, we have demonstrated native mass spectrometry imaging, that is spatial profiling of folded intact proteins and protein assemblies in thin tissue sections of mouse liver and mouse brain^75^ (see Figure 9D). Furthermore, the benefit of incorporating TWIMS into the mass spectrometry workflow is demonstrated in the measurement of CCS for a range of folded intact proteins directly from mouse brain tissue. The CCS of 5 different intact protein species ubiquitin (5+), β‐thymosin 4 (4+), and β‐thymosin 10 (4+) and three further unidentified protein ions of m/z 1187 (4+), 1184 (4+), and 1567 (10+) were calculated to be 1047 ± 8, 733 ± 2, 796 ± 2, 728 ± 6, 772 ± 5, and 2453 ± 17 Å respectively.^75^ The calculated CCS of ubiquitin was in agreement with that of the purified protein.

## 7 PERSPECTIVE

In situ protein analysis is developing along two avenues: imaging of intact proteins within thin tissue sections and microbial analysis. For the former, the native LESA approach presents a number of exciting opportunities, namely probing protein tertiary and quaternary structure directly from biological substrates and investigating protein ligand binding interactions. The integration of ion mobility separation with imaging workflows is key in this regard. Whilst native LESA mass spectrometry imaging allows the analysis of folded proteins and protein complexes in a spatially defined manner, protein tertiary (and quaternary) structure could be probed via CCS measurements within the same experiment.

For microbial analysis, one of the priorities would be the application of intact protein analysis by mass spectrometry to a greater range of clinically relevant species, seeking applications in biofilm studies, pathogen‐host interactions, and antibiotic development. An expansion of the range of observed proteins would be greatly beneficial; as outlined above, rapid separation methods such as FAIMS may provide one possible avenue to this end and should therefore be explored alongside any new developments in ion mobility spectrometry. Targeted analysis of proteins relevant to pathogenesis and antibiotic resistance should then become possible.
